# Inflammatory Cytokine Gene Expression in Mesenteric Adipose Tissue during Acute Experimental Colitis

**DOI:** 10.1371/journal.pone.0083693

**Published:** 2013-12-26

**Authors:** W. Conan Mustain, Marlene E. Starr, Joseph D. Valentino, Donald A. Cohen, Daiki Okamura, Chi Wang, B. Mark Evers, Hiroshi Saito

**Affiliations:** 1 Markey Cancer Center, University of Kentucky, Lexington, Kentucky, United States of America; 2 Department of Surgery, University of Kentucky, Lexington, Kentucky, United States of America; 3 Department of Microbiology, Immunology, and Molecular Genetics, University of Kentucky, Lexington, Kentucky, United States of America; 4 Department of Biostatistics, University of Kentucky, Lexington, Kentucky, United States of America; 5 Department of Physiology, University of Kentucky, Lexington, Kentucky, United States of America; Charité, Campus Benjamin Franklin, Germany

## Abstract

**Background:**

Production of inflammatory cytokines by mesenteric adipose tissue (MAT) has been implicated in the pathogenesis of inflammatory bowel disease (IBD). Animal models of colitis have demonstrated inflammatory changes within MAT, but it is unclear if these changes occur in isolation or as part of a systemic adipose tissue response. It is also unknown what cell types are responsible for cytokine production within MAT. The present study was designed to determine whether cytokine production by MAT during experimental colitis is depot-specific, and also to identify the source of cytokine production within MAT.

**Methods:**

Experimental colitis was induced in 6-month-old C57BL/6 mice by administration of dextran sulfate sodium (2% in drinking water) for up to 5 days. The induction of cytokine mRNA within various adipose tissues, including mesenteric, epididymal, and subcutaneous, was analyzed by qRT-PCR. These adipose tissues were also examined for histological evidence of inflammation. The level of cytokine mRNA during acute colitis was compared between mature mesenteric adipocytes, mesenteric stromal vascular fraction (SVF), and mesenteric lymph nodes.

**Results:**

During acute colitis, MAT exhibited an increased presence of infiltrating mononuclear cells and fibrotic structures, as well as decreased adipocyte size. The mRNA levels of TNF-α, IL-1β, and IL-6 were significantly increased in MAT but not other adipose tissue depots. Within the MAT, induction of these cytokines was observed mainly in the SVF.

**Conclusions:**

Acute experimental colitis causes a strong site-specific inflammatory response within MAT, which is mediated by cells of the SVF, rather than mature adipocytes or mesenteric lymph nodes.

## Introduction

In addition to its role in energy storage, adipose tissue is widely recognized as a dynamic metabolic organ involved in the regulation of immunity and inflammation. Distributed throughout the body in distinct depots, adipose tissue exhibits specific inflammatory profiles depending on location [1], [2]. Intra-abdominal adipose tissue has been implicated in the pathogenesis of several gastrointestinal diseases including fatty liver disease [3], acute pancreatitis [4], colon cancer [5], and inflammatory bowel disease (IBD) [6]. Increased cytokine expression from mesenteric adipose tissue (MAT) adjacent to the affected bowel in Crohn's disease suggests a potential causal role of MAT in the pathophysiology of this disease [7], [8].

Recent studies using animal models of IBD have shown inflammatory changes within MAT, such as elevated levels of inflammatory cytokines, infiltration of immune cells, and increased release of free fatty acids [9], [10], [11], [12], [13]. While these findings in animal studies suggest a commonality with Crohn's disease, there are several questions that remain unanswered. Specifically, it is unclear whether adipose tissue inflammation during experimental colitis is specific to the mesentery, or rather, part of a generalized systemic response. Batra *et al.* recently reported their study on mice with experimental colitis showing that MAT, but not subcutaneous adipose tissue (SAT), was infiltrated with inflammatory cells and released increased levels of cytokines [14]. We have previously demonstrated strong expression of inflammatory cytokine genes in the epididymal adipose tissue (EAT) of mice during systemic inflammation induced by bacterial endotoxin lipopolysaccharide (LPS) [15], [16]. However, no previous studies have compared MAT and EAT, two sources of visceral fat, in parallel during acute colitis.

Furthermore, adipose tissue is a heterogenous tissue containing not only mature adipocytes, but also a dense network of blood vessels, nerves, undifferentiated pre-adipocytes, and immune cells in a connective tissue matrix. It is unknown what cell types are responsible for the increased expression of inflammatory cytokines in adipose tissues during colitis.

In the current study, we used a murine model of experimental colitis to determine whether cytokine expression in MAT during colitis is a depot-specific phenomenon or simply a reflection of systemic adipose tissue inflammation. Additionally we examined whether or not the mature adipocytes are the primary cellular source of cytokine expression in adipose tissue during colitis.

## Materials and Methods

### Animals

Young adult (6 month-old) C57BL/6 male mice were obtained from colonies of the National Institute on Aging (Bethesda, MD). Although 6–8 week-old mice are frequently used in IBD models, 6 month-old mice were used in this study because they represent sexually mature, young adults [17] with more adipose tissue than their younger, immature counterparts. Prior to experiments, all mice were maintained for at least 7 days in an environment under controlled temperature (21–23°C), humidity (30–70%), and lighting (14 hours light/10 hours dark) with free access to drinking water and chow (Rodent Diet No. 2500, LabDiet, St. Louis, MO). All animal procedures were approved by the Institutional Animal Care and Use Committee at the University of Kentucky.

### Experimental Colitis

Experimental colitis was induced in mice by adding dextran sulfate sodium (DSS),(molecular weight 40,000–50,000; USB, Cleveland, OH) to drinking water at a concentration of 2% for up to 5 days, after which the water was replaced with regular drinking water without DSS. Mice were sacrificed (n = 4–5 per time point) at Day 0 (immediately prior to DSS exposure), Day 3, Day 7 (2 days after withdrawal of DSS), and Day 14 (9 days after withdrawal of DSS) of the experimental period. Body weight was recorded daily between 11am and 2pm. At the time of sacrifice, mice were anesthetized by isoflurane inhalation (2–5% in air) and blood (600–800 µL) was collected slowly from the inferior *vena cava* (IVC) using a heparin-coated syringe with a 25-gauge needle. For tissue RNA analysis, the IVC was cut and the entire vasculature was perfused with physiological saline (0.9% NaCl) through the cardiac ventricles. Whole colons, mesenteric adipose tissue (MAT), epididymal adipose tissue (EAT), and inguinal subcutaneous adipose tissue (SAT) were subsequently collected, flash frozen in liquid nitrogen, and stored at −80°C until use. For histological analysis, tissues were collected without circulatory perfusion, placed in 10% neutral buffered formalin for 24 hours, and embedded in paraffin.

### Clinical Scoring of Colitis

The clinical severity of colitis was assessed by calculation of disease activity index (DAI), as previously described [18]. Briefly, DAI is based on 3 parameters: changes in body weight, presence of stool blood, and stool consistency. Weight change was based on initial body weight the day prior to DSS exposure. The presence of stool blood was assessed by ColoScreen® fecal occult blood test cards (Helena Laboratories, Beaumont, TX). Mice are given a score (0–4) for each parameter and the DAI is calculated as the average of the three scores.

### Histology and Disease Scoring

Entire colons were measured lengthwise, opened longitudinally, and Swiss-rolled prior to formalin fixation and paraffin embedding. Tissue sections (5-µm) were stained with hematoxylin and eosin (H&E) and evaluated microscopically. Each segment of the colon (proximal, middle, and distal) was given a score (grade 0 to 4) based on the criteria described by Berg et al. [19]. The summation of these scores provided a total colonic disease score per mouse. The disease scores could range from 0 (no change in any segment) to a maximum of 12 (grade 4 lesions in all three segments). Photomicrographs were taken using a Nikon Eclipse E200 microscope and Nikon Digital Sight DS-U3/DSFi1 digital camera system with NIS Elements F3.2 Imaging Software (Nikon Inc., Melville, NY).

To evaluate changes in adipocyte size during colitis, adipocytes were counted in 3 independent microscopic fields **(×400)** of MAT sections from each mouse at Day 0 and 7 (5 mice per group). Due to the presence of fibrotic areas in MAT of colitis mice, cell counts were made from adipocyte-rich areas of the tissue sections and a proportional area in control slides were evaluated in parallel.

### RNA Isolation from Whole Tissues

Total RNA was isolated from tissues according to our previously described protocol [16]. RNA was preserved in nuclease-free water and stored at −80°C until use. RNA concentration of each sample was determined by a spectrophotometer (DU730 Life Science UV/Vis, Beckman Coulter, Indianapolis, IN). The quality of each RNA sample was confirmed by agarose gel electrophoresis followed by visualization of 28 s and 18 s rRNA bands by staining with ethidium bromide.

### Plasma cytokine analysis

At the time of sacrifice, collected blood was centrifuged at 2000× g for 10 minutes. Plasma supernatants were collected, aliquotted into 0.5 mL tubes, and stored at −80°C until use. Concentrations of plasma TNF-α and IL-6 were measured by Mouse ELISA Kits (Thermo Scientific, Rockford, IL) according to the manufacturer's protocol. The minimum detection limit of the TNF-α and IL-6 ELISA kit was 9 pg/mL and 7 pg/mL, respectively. Results were determined by BioTek ELx800 plate reader with Gen 5 software.

### Adipose Tissue Fraction Separation

For RNA analysis of individual adipose tissue fractions, mice were anesthetized and perfused as described above. Whole mesenteries from 2 to 4 mice sacrificed simultaneously were pooled, minced, and digested by Type I Collagenase (1 mg/mL, Sigma, St. Louis, MO) in 4-ml of isolation buffer (2% BSA-HBSS +5 mM glucose) for 1 hour at 37°C, and passed through a nylon strainer with 100 µm pore-size (BD Falcon, Franklin Lakes, NJ). Mesenteric lymph nodes (MLN) were manually collected from the strainer (4 per mouse) as they were not digested by collagenase. The strained material was centrifuged at 450× g for 10 minutes. Floating adipocytes were collected and the remaining precipitated stromal vascular fraction (SVF) was resuspended in isolation buffer. Each of the three separated fractions (MLN, adipocytes, and SVF) was re-centrifuged and isolation buffer was discarded. RNA was isolated from each fraction using RNeasy Lipid Tissue Mini Kit (Qiagen, Valencia, CA) according to the manufacturer's protocol. RNA concentration was measured by NanoDrop ND-1000 (Thermo Fisher Scientific, Waltham, MA) and RNA integrity was confirmed by Agilent 2100 Bioanalyzer (Agilent Technologies, Santa Clara, CA).

### Analysis of cytokine mRNA levels by qRT-PCR

Real-time quantitative reverse transcriptase-polymerase chain reaction (qRT-PCR) was performed to assess cytokine mRNA levels in purified RNA samples from tissues. Equivalent amounts of RNA were reverse-transcribed into cDNA using SuperScript® III First-Strand Synthesis SuperMix (Life Technologies, Grand Island, NY) according to the manufacturer's protocol. Primers for PCR were designed using the Universal ProbeLibrary (Roche Applied Science, Indianapolis, IN) and purchased from Integrated DNA Technologies (Coralville, IA). The qRT-PCR reaction was performed on Roche Light Cycler 480. Each 20 µL reaction was performed in duplicate and contained 5 µL of diluted cDNA, 5′ and 3′ primers (400 nM), dual-labeled Universal ProbeLibrary Probes (100 nM), and 10 µL LightCycler® 480 Probes Master mix. Target gene expression was normalized to hypoxanthine-guanine phosphoribosyl transferase (HPRT) expression as an endogenous control, and fold change was calculated as 2^−(ΔΔC^_T_^)^, using the mean ΔC_T_ of the Day 0 group as a calibrator. Target gene expression levels from adipose tissue fractions were adjusted by the percentage of the total RNA yield obtained from each fraction.

### Statistical Analysis

Statistical analysis was performed using R 2.14.0 (http://www.R-project.org/) and SAS 9.2 (SAS Institute, Cary NC). A paired t-test was used to compare body weight at each time point versus the baseline. A one-way ANOVA model was used to compare colon length measured at different days, and contrasts were subsequently generated for pairwise comparisons. A similar approach was used to analyze the expression of inflammatory cytokines from MAT at multiple time points. A Kruskal-Wallis test was used to compare DAI measured at different days. Pairwise post-hoc analysis was carried out using the Wilcoxon rank-sum test. A similar approach was used to assess histologic severity score measured at different days. Linear mixed models were used for comparison of inflammatory cytokine expressions among tissue types and between days of measurement. Similar approaches were used for comparison of cell-type markers and cytokine expression among cellular fractions and between days of measurement. All the reported P values have been adjusted for multiple comparisons based on the Holm's procedure. Statistical significance was defined as P<0.05.

## Results

### Development and Progression of Experimental Colitis

**Figure 1:**
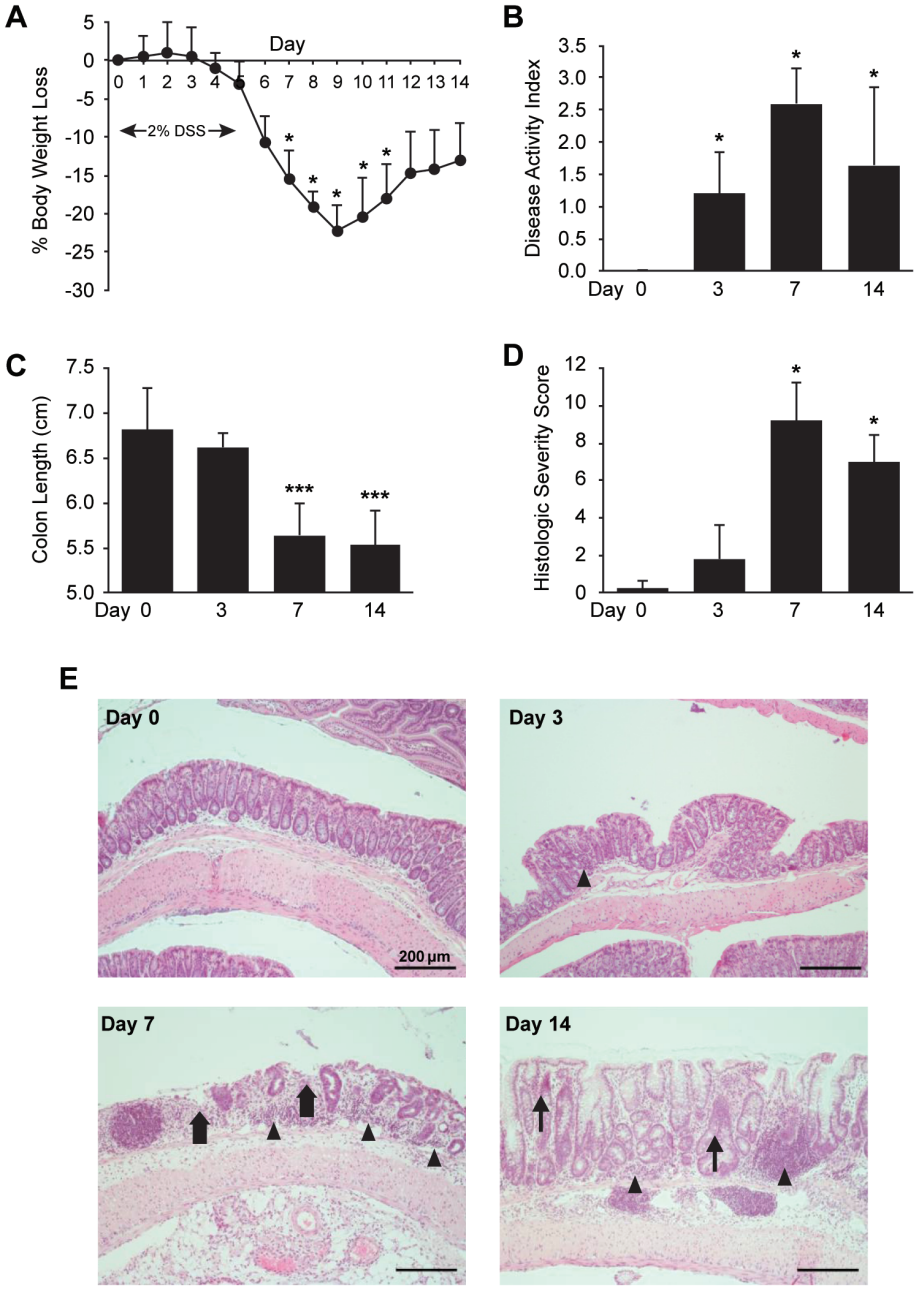
Development and progression of experimental colitis. Male, 6 month-old C57BL/6 mice (*n* = 20) were exposed to 2% DSS in drinking water for up to 5 days. Mice were sacrificed at Day 0, 3, 7, and 14 of the experimental period (*n* = 5 per time point). (A) Daily body weight of mice sacrificed at Day 14; mice sacrificed at earlier time points followed a similar pattern. *P<0.05 compared to initial body weight on Day 0. (B) Disease Activity Index (DAI) was calculated at the time of sacrifice based on weight loss, stool blood, and stool consistency. (C) Colons were excised and measured lengthwise. (D) H&E stained sections were graded for Histologic Severity Score. (E) Representative images of colon histology on Day 0, 3, 7, and 14 (original magnification × 100; arrowhead  =  mononuclear infiltrates, thick arrow  =  mucosal ulceration, thin arrow  =  epithelial hyperplasia). Data represent mean ± SD, ***P<0.001, *P<0.05 vs. Day 0.

To characterize the development and progression of experimental colitis, mice were weighed daily and assessed for clinical and histologic evidence of colitis at four different time points during the experimental period (Day 0, 3, 7, and 14). Weight loss was noted on Day 4, and became statistically significant by Day 7 (*P*<0.05 vs. initial body weight). Peak weight loss was noted at Day 9, while some recovery of body weight occurred by Day 12 (Fig. 1A). The earliest clinical evidence of acute colitis was bloody stool noted in some mice at Day 3, resulting in a significant increase in Disease Activity Index (DAI) compared to animals sacrificed at Day 0. Of the time points studied, DAI was most severe at Day 7, but remained elevated through Day 14 (Fig. 1B). DAI at Day 14 was predominantly attributable to loose stool and weight loss, as bloody stools had resolved in all but one animal. Significant colon shortening was not seen until Day 7 but persisted through Day 14, despite improvement in DAI and body weight (Fig. 1C).

Histological evidence of colitis was present by Day 3 (Fig. 1D, E), with Grade 1 lesions (mild inflammation, comprised mainly of mononuclear cells, with little epithelial damage) noted in multiple colon segments. Histologic severity scores were highest at Day 7, with Grade 4 lesions (severe inflammation, mononuclear cell infiltration, mucosal ulceration) seen in the middle and distal colons of all mice; less severe inflammation (Grade 2 and 3 lesions) was identified in the proximal colon. Despite partial recovery of body weight and the cessation of rectal bleeding, mice sacrificed at Day 14 showed evidence of ongoing severe inflammation, and a significantly elevated mean histologic severity score. Although mucosal regeneration was seen at this time point, the presence of transmural inflammation and mononuclear cell infiltration, along with focal epithelial hyperplasia and persistent colon shortening was consistent with ongoing chronic inflammation.

### Adipose Tissue Inflammation during Acute Colitis

**Figure 2:**
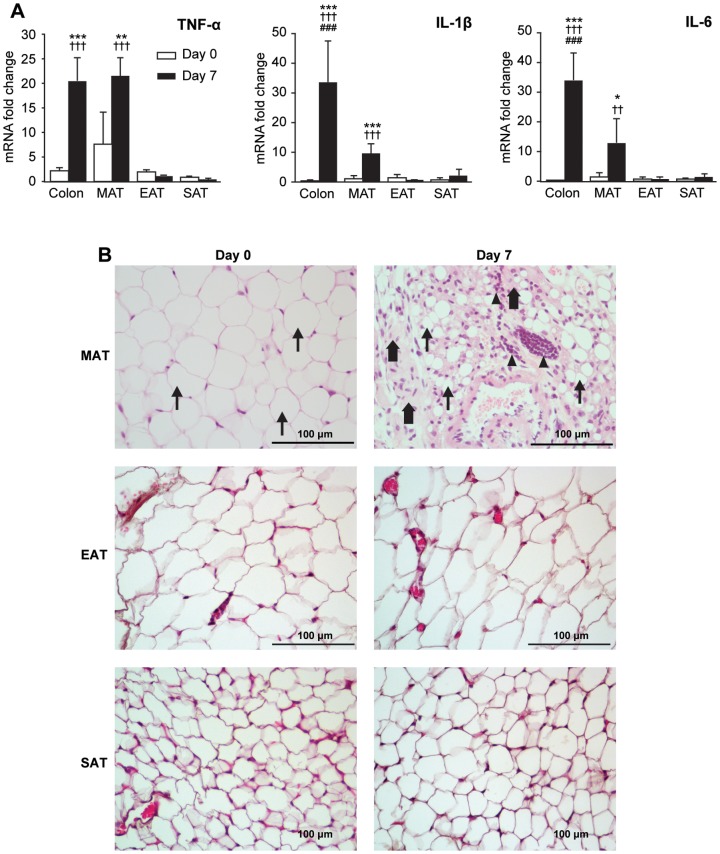
Adipose tissue inflammation during acute colitis. (A) Colons and adipose tissue [mesenteric (MAT), epididymal (EAT), and subcutaneous (SAT)] from mice sacrificed at Day 0 and Day 7 (*n* = 4 per timepoint) were analyzed for relative mRNA expression of TNF-α, IL-1β, and IL-6. Target gene expression was normalized to HPRT gene expression; fold change was calculated relative to mean SAT expression at Day 0. Data represent mean ± SD. ***P<0.001, **P<0.01, *P<0.05 vs. Day 0 of the same tissue; †††*P*<0.001, ††*P*<0.01 vs. EAT and SAT at Day 7; ###*P*<0.001 vs. MAT at Day 7. (B) H&E stained sections of formalin-fixed MAT, EAT, and SAT from Day 0 and Day 7 of experimental colitis were compared (*n* = 5 per time point). Representative images are shown (original magnification × 400; arrowhead  =  mononuclear infiltrates; thick arrow  =  fibrotic connective tissue; thin arrow  =  adipocytes).

After characterizing the progression of experimental colitis, we next determined which adipose tissues exhibited increased expression of inflammatory cytokines during acute colitis. Transcript levels of several cytokines were compared among the colon and mesenteric (MAT), epididymal (EAT), and subcutaneous (SAT) adipose tissue at Day 0 and Day 7 of experimental colitis. As shown in Figure 2A, TNF-α, IL-1β, and IL-6 mRNA expression was significantly induced in the colon during acute colitis (*P*<0.001, for each cytokine). Among the adipose tissues examined, only MAT showed significant induction of inflammatory cytokines; mRNA expression of TNF-α (*P* = 0.003), IL-1β (*P*<0.001), and IL-6 (*P* = 0.038) was significantly increased at Day 7 vs. Day 0. No induction of these cytokines was seen in EAT or SAT at Day 7. During acute colitis, TNF-α expression in the MAT was equivalent per unit of RNA to that of the colon, and significantly greater (*P*<0.001) than the other adipose tissue depots. MAT mRNA levels of IL-1β (*P*<0.001) and IL-6 (*P* = 0.002) were also significantly higher than that of EAT or SAT during acute colitis, although the colon showed the highest expression of these cytokines. Plasma levels of TNF-α and IL-6, measured by ELISA, remained undetectable (<9 pg/mL) throughout the experimental period in nearly all animals (data not shown).

To investigate the histologic changes associated with MAT inflammation during acute experimental colitis, H&E stained sections of formalin-fixed whole mesenteries from animals sacrificed at Day 0 and Day 7 of experimental colitis were compared. At the time of collecting adipose tissues, we did not notice any colitis-induced macroscopic difference such as creeping fat which was observed in another experimental colitis model [20] (data not shown). Microscopic observation revealed that, during acute colitis, MAT exhibited intense mononuclear cell infiltration, the appearance of fibrotic structures within the tissue, and a decrease in adipocyte size relative to Day 0 (Fig. 2B). Compared to Day 0, number of mesenteric adipocytes per microscopic field were 2.7-fold higher (22.8±4.63 vs 62.0±11.77, p<0.001), indicating that the average size of adipocytes in MAT significantly decreased during acute colitis. In contrast to MAT, both EAT and SAT demonstrated no significant histologic change with acute colitis (Fig. 2B).

### Time-course of Mesenteric Adipose Tissue Cytokine Expression

**Figure 3:**
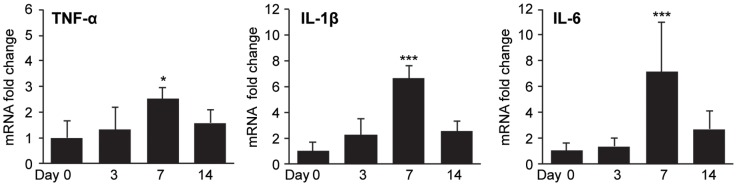
Time-course of mesenteric adipose tissue cytokine expression. Mice were sacrificed at Day 0, 3, 7, and 14 of experimental colitis (*n* = 5 per time point). (A) MAT was analyzed for relative mRNA expression of TNF-α, IL-1β, and IL-6. Target gene expression was normalized to HPRT expression; fold change was calculated relative to mean expression at Day 0. Data represent mean ± SD, ***P<0.001, *P<0.05 vs. Day 0.

After determining that MAT inflammation during acute colitis is depot-specific, we next determined how MAT-derived cytokine expression changed throughout the progression of experimental colitis. To this end, we analyzed expression of TNF-α, IL-1β, and IL-6 mRNA from MAT at multiple time points. Similar to our multi-tissue analysis, significant increases in TNF-α, IL-1β, and IL-6 mRNA expression were seen at Day 7 (*P* = 0.024, <0.001, <0.001, respectively). However, no statistically significant induction of these cytokines was seen at Day 3 or Day 14 (Fig. 3). EAT and SAT were also analyzed at these additional time points with no significant induction of TNF-α, IL-1β, and IL-6 mRNA (data not shown). Day 7 mRNA levels relative to Day 0 were similar to the multi-tissue analysis (Fig. 2A) for all cytokines.

### Mesenteric Adipose Tissue Cytokine Production Occurs Primarily in the Stromal Vascular Fraction

**Figure 4:**
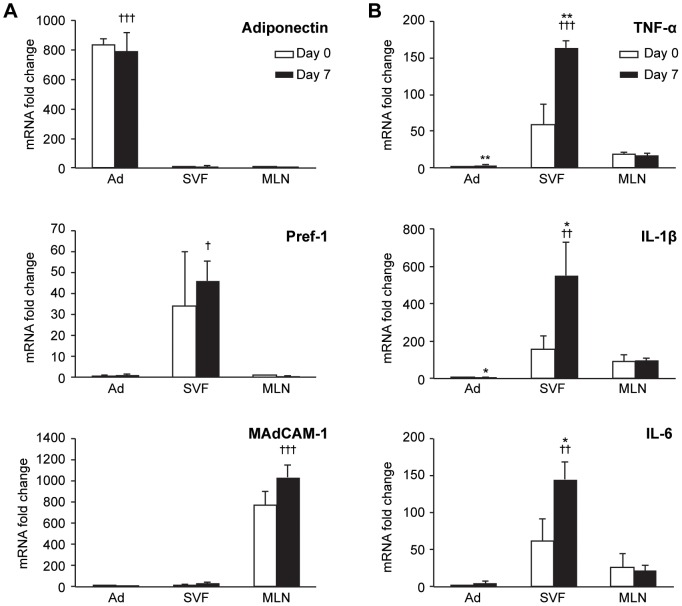
Cytokine expression from MAT adipocytes (Ad), stromal vascular fraction (SVF), and mesenteric lymph nodes (MLN) during acute experimental colitis. (A) The adipocyte-specific hormone, Adiponectin; the preadipocyte marker, Pref-1; and the lymph node marker, MAdCAM-1, were used to confirm successful separation of Ad, SVF, and MLN, respectively. Target gene expression was normalized to HPRT expression; fold change was calculated relative to MLN expression at Day 0 (for Adiponectin and Pref-1) or Ad expression at Day 0 (for MAdCAM-1). (B) TNF-α, IL-1β, and IL-6 expression was compared between Ad, SVF, and MLN at Day 0 and Day 7. Target gene expression was normalized to HPRT expression and adjusted for RNA yield; fold change was calculated relative to Ad expression at Day 0. Data represent mean ± SD, ***P<0.001, **P<0.01, *P<0.05 vs. Day 0 of the same fraction; †††*P*<0.001, ††*P*<0.01 vs. other fractions at Day 7.

Having shown adipose-associated cytokine induction to be specific to MAT and limited to the acute phase of experimental colitis, we next determined the cellular source of cytokine expression. Whole mesenteries from mice at Day 7 of DSS-induced colitis were digested with collagenase to isolate mature adipocytes from the stromal vascular fraction (SVF) and mesenteric lymph nodes (MLN), allowing independent analysis of each fraction. Successful separation of cellular fractions was confirmed by the expression of specific cell-type markers including adiponectin (a marker for adipocytes), preadipocyte factor-1 (Pref-1, a marker for preadipocytes of the SVF), and mucosal addressin cell adhesion molecule-1 (MAdCAM-1, a marker for high-endothelial venules within lymph nodes). As shown in Fig. 4A, adiponectin, Pref-1, and MAdCAM-1 were exclusively expressed in adipocyte fraction, SVF, and MLN, respectively, confirming successful isolation of three distinct cellular fractions from the mesentery.

We then evaluated the expression of TNF-α, IL-1β, and IL-6 from each fraction to determine their relative contributions to mesenteric cytokine expression during acute colitis. Colitis-induced upregulation of TNF-α, IL-1β, and IL-6 occurred predominantly in the SVF (Figure 4B). At Day 7 of acute colitis, the amount of TNF-α, IL-1β, and IL-6 expressed in the SVF was significantly increased relative to Day 0 (*P* = 0.005, 0.031, and 0.038, respectively). Furthermore, for each of the cytokines analyzed, the majority of the expression during acute colitis occurred in the SVF. Day 7 expression of TNF-α in SVF was 45.3-fold and 9.5-fold greater than that in adipocytes or MLN, respectively (*P*<0.001 for each). IL-1β expression was 115.8-fold (*P* = 0.003) and 5.7-fold (*P* = 0.005) greater in SVF than in adipocytes or MLN, respectively. IL-6 was also significantly higher in SVF than in adipocytes or MLN (30.0-fold and 6.8-fold, respectively). A minimal induction of these cytokines was also seen in the adipocyte fraction at Day 7, though the levels were considerably lower than that of the SVF. Expression of TNF-α, IL-1β, and IL-6 in MLN did not change with acute colitis. These data clearly demonstrate that the increased expression of TNF-α, IL-1β, and IL-6 observed in MAT during acute colitis is predominantly due to increased expression of these cytokines in the SVF, rather than mature adipocytes or MLN.

## Discussion

In the present study, we investigated the induction of inflammatory cytokine gene expression in MAT during DSS-induced acute experimental colitis in mice. Although inflammation in MAT is a well-documented phenomenon in Crohn's disease, it was only recently that inflammatory changes within MAT were demonstrated in several animals models of experimental colitis induced by either 2,4,6-trinitrobenzene sulfonic acid (TNBS) [10], [12], [21], 2,4-dinitrobenzene sulfonic acid (DNBS) [20], or DSS [14], [22]. In these studies, however, MAT inflammation was investigated in isolation or in comparison to SAT [14] but not to another source of visceral fat such as EAT. Our previous work identifying EAT as a major source of cytokine expression during systemic inflammation [15], [16] prompted us to conduct the current study to determine if MAT inflammation was depot-specific or part of a generalized, systemic or visceral inflammatory response during acute colitis. Our results demonstrate that, while MAT showed strong inflammatory cytokine gene expression and histological changes during acute colitis, similar inflammatory response was completely absent in both EAT and SAT, disputing the possibility of a generalized adipose tissue response. Thus, we conclude that cytokine gene expression in MAT is a depot-specific phenomenon during acute colitis. Such site-specific inflammatory responses within the mesentery suggest a possible paracrine role for mesenteric-derived cytokines in mediating intestinal inflammation.

To increase the sensitivity and specificity of our analyses, we measured cytokine mRNA levels rather than protein levels. To eliminate the risk of tissue contamination by circulating blood cells, we performed circulatory perfusion with physiological saline before tissue collection. Thus our findings of elevated cytokine gene expression are specific to the adipose tissues and are not derived from circulating inflammatory cells. Although the current study was limited to cytokine mRNA expression, we speculate that the same cytokines are actually produced and released from the adipose tissues. A recent study by Batra et al. has indeed demonstrated that isolated MAT from mice with colitis released these cytokines ex vivo [14].

In contrast to our present study of acute colitis in which inflammatory responses are limited to the colon and MAT, several previous studies on chronic colitis have reported more generalized inflammation. For example, Li et al. [23] reported elevated protein levels of IL-1β and IL-12 in MAT, SAT, liver, and colon in mice with DSS-induced chronic colitis, which suggests presence of systemic inflammation. Teixeira et al. [24] demonstrated neutrophil infiltration of EAT in mice with chronic colitis but no increase in TNF-α or IL-6 expression; in this study MAT was not analyzed. These studies illustrate that prolonged inflammation can induce inflammatory changes in multiple tissues and highlight the importance of distinguishing a local tissue reaction from a systemic response.

We did not detect elevated plasma concentrations of TNF-α or IL-6 in the 2% DSS model used in this study. Elevated plasma IL-6 was previously reported in acute colitis induced by 5% DSS, though TNF-α was still undetectable even in this more severe model [25]. Plasma IL-1β also does not increase in acute colitis induced by 3% DSS [26]. Taken together, the degree of systemic inflammation during DSS-induced acute colitis appears to be minimal.

MAT from human Crohn's disease patients exhibits infiltration of inflammatory cells, perivascular inflammation, stromal fibrosis, reduced adipocyte size, and elevated levels of inflammatory cytokines and adipocyte-specific hormones [7], [8], [22], [27], [28]. In the present study of MAT inflammation in mice with DSS-induced colitis, we observed histological changes and induction of inflammatory cytokines that are similar to those seen in the mesentery of Crohn's patients. Histologic changes were not seen in EAT or SAT with acute colitis in our study. This regional specificity is also similar to human IBD, in which adipose tissue inflammation appears localized to the mesentery [29], [30]. These findings highlight the relevance of this animal model to the human condition of MAT inflammation in IBD.

The administration of DSS to C57BL/6 mice results in a reproducible acute experimental colitis which progresses in chronicity after only a single exposure [31]. DSS is directly toxic to gut epithelial cells and disrupts the integrity of the mucosal barrier through a non-inflammatory dropout of crypts, prior to the development of intestinal inflammation [18], [32]. Additionally, treatment with DSS was recently shown to significantly increase the rate of bacterial translocation to MAT and mesenteric lymph nodes in mice [22]. Because defects in epithelial integrity and early bacterial translocation, occurring prior to the onset of colonic inflammation, have been postulated as possible etiologies of colonic inflammation in Crohn's disease [33], [34], this colitis model appears to provide insight into the timing of MAT inflammation relative to colonic inflammation after early disruption of the mucosal barrier. Importantly, our examination of MAT at Day 3 of DSS exposure revealed no induction of inflammatory cytokines, despite clinical and histologic evidence of colitis at this time point. While this finding does not exclude the possibility of MAT inflammation as a precursor to colonic inflammation in Crohn's disease, it does not appear that disruption of epithelial integrity alone is sufficient to cause MAT inflammation prior to the onset of acute colitis. Additionally, while colonic inflammation persisted at Day 14 (Fig. 1), cytokine expression in MAT was elevated only during the peak phase (Day 7) of acute colitis (Fig. 3). It is possible that cytokine expression in MAT during acute colitis contributes to the activation of the acquired immune response and drives the transition to the chronic phase of colitis, which is more likely to be T-cell mediated [35].

Adipose tissue consists of mature adipocytes as well as other cell types including vascular endothelial cells, preadipocytes, macrophages, and inflammatory cells, which are all capable of producing inflammatory cytokines. In obese humans, non-adipocyte components of adipose tissue have been shown to be a more significant source of inflammatory cytokines than adipocytes [36], [37]. Recent work from our laboratory also found that cells in the SVF and not adipocytes, are the major source of expression of TNF-α, IL-1β, IL-6, and multiple other inflammatory and pro-coagulant genes within EAT during LPS-induced systemic inflammation [16]. The current study also demonstrates that induction of inflammatory cytokines occurs predominantly in the SVF of MAT during acute colitis, with no increased expression in mesenteric lymph nodes (MLN) and minimally increased expression in the adipocyte fraction. When adjusted for the greater proportion of MAT mRNA obtained from the SVF relative to adipocytes, the differential contribution of these two fractions to MAT-derived cytokine expression is readily apparent. Expression of all three measured cytokines was at least 30-fold higher in the SVF than in the adipocyte fraction, indicating that SVF cells are predominantly responsible for the observed increase in expression from whole MAT during acute colitis. Increased expression of cytokines in the adipocyte fraction could be due to contaminating SVF cells within this fraction due to the adhesive property of adipocytes, making complete separation difficult. The specific cell type within the SVF that accounts for the greatest cytokine expression was not identified in this study. Induction within this fraction could be due to infiltrating mononuclear cells, adipose tissue macrophages, vascular endothelial cells, or preadipocytes. Preadipocytes, which are specific to the SVF, are known to express high levels of IL-6 in response to LPS [36] and may represent the major cell type responsible for MAT cytokine expression during acute colitis.

In summary, during DSS-induced acute colitis in mice, the inflammatory response in MAT occurs after the onset of colonic inflammation and subsides with progression to chronic colitis. This response is specific to the mesentery and does not occur in other adipose tissue depots such as EAT and SAT that are known to produce cytokines during other inflammatory disease states. Furthermore, within the MAT, the cells of the SVF are primarily responsible for cytokine expression. Although the role of adipose tissue inflammation in Crohn's disease remains unclear, MAT is increasingly recognized as an active participant in the inflammatory response in patients suffering with this disease [38], [39]. The present study provides important new information related to the specificity, timing, and source of MAT-derived cytokine expression in a murine model of colitis.
